# Genetic Variation in Acid Ceramidase Predicts Non-completion of an Exercise Intervention

**DOI:** 10.3389/fphys.2018.00781

**Published:** 2018-06-29

**Authors:** Lauren S. Lewis, Kim M. Huffman, Ira J. Smith, Mark P. Donahue, Cris A. Slentz, Joseph A. Houmard, Monica J. Hubal, Eric P. Hoffman, Elizabeth R. Hauser, Ilene C. Siegler, William E. Kraus

**Affiliations:** ^1^Department of Obstetrics and Gynecology, Duke University School of Medicine, Durham, NC, United States; ^2^Duke Molecular Physiology Institute, Duke University School of Medicine, Durham, NC, United States; ^3^Division of Rheumatology and Immunology, Department of Medicine, Duke University School of Medicine, Durham, NC, United States; ^4^Division of Cardiology, Department of Medicine, Duke University School of Medicine, Durham, NC, United States; ^5^Human Performance Laboratory, East Carolina University, Greenville, NC, United States; ^6^Children's Genetic Medical Research Center, Children's National Medical Center, Washington, DC, United States; ^7^Cooperative Studies Program-Epidemiology Center Durham, Veterans Administration Medical Center, Durham, NC, United States; ^8^Division of Behavioral Medicine, Department of Psychiatry, Duke University School of Medicine, Durham, NC, United States

**Keywords:** STRRIDE, metabolism, ceramide, exercise adherence, behavioural lifestyle interventions

## Abstract

Genetic variation is associated with a number of lifestyle behaviours; it may be associated with adherence and individual responses to exercise training. We tested single nucleotide polymorphisms (SNPs) in the acid ceramidase gene (*ASAH1*) for association with subject adherence and physiologic benefit with exercise training in two well-characterised randomised, controlled 8-month exercise interventions: STRRIDE I (*n* = 239) and STRRIDE II (*n* = 246). Three *ASAH1* non-coding SNPs in a linkage disequilibrium block were associated with non-completion: rs2898458(G/T), rs7508(A/G), and rs3810(A/G) were associated with non-completion in both additive (OR = 1.8, 1.8, 2.0; *P* < 0.05 all) and dominant (OR = 2.5, 2.6, 3.5; *P* < 0.05 all) models; with less skeletal muscle *ASAH* expression (*p* < 0.01) in a subset (*N* = 60); and poorer training response in cardiorespiratory fitness (peak VO_2_ change rs3810 *r*^2^ = 0.29, *P* = 0.04; rs2898458 *r*^2^ = 0.29, *P* = 0.08; rs7508 *r*^2^ = 0.28, *p* = 0.09); and similar in direction and magnitude in both independent exploratory and replication studies. Adherence to exercise may be partly biologically and genetically moderated through metabolic regulatory pathways participating in skeletal muscle adaptation to exercise training.

## Introduction

Many of the health benefits of exercise are mediated by metabolic adaptations in skeletal muscle, and increases in cardiorespiratory fitness. As detailed in the Physical Activity Guidelines Advisory Committee Report the benefits of exercise are substantial (DHHS, 2008) however, perhaps the major clinical issue confronting the use of exercise training as a therapeutic option is how to get individuals to initiate an exercise training program and—following that—how to maintain it. Common wisdom holds that adherence issues are primarily related to neurobehavioural and social issues, and that barriers can be identified and addressed using behavioural approaches. Although most attention regarding adherence to lifestyle interventions has traditionally focused on psychosocial behavioural factors, it is conceivable that there also exist biological and genetic factors determining whether individuals maintain an exercise program once initiated.

An additional issue complicating the use of exercise training as a therapeutic option is the variability in training responses. Conventional wisdom holds that more exercise is better, and that exercise is beneficial for everyone for a myriad of health benefits. Rather, even when controlling for adherence, there is a range of health responses to any given exercise program for any given health parameter (e.g., fitness, blood lipids, insulin sensitivity, blood pressure control, even weight change). In sum, not all individuals respond in a similar—or even in a favourable manner—to an exercise program (Bouchard et al., 2012). Such variation in biological response to a given exercise exposure is heritable; with heritability estimates ranging from 29 to 70%. This suggests a large fraction of the exercise response is moderated by genetic factors. These observations raised the prospect that genetic classifiers might be assembled to predict the variability in responses to exercise.

Genes of metabolic pathways active in skeletal muscle are associated with exercise performance; (Bouchard et al., 2011) and many of the physiologic effects of exercise training result from the adaption of skeletal muscle mitochondria (Duscha et al., 2005; Huffman et al., 2014). Thus, genes involved in skeletal muscle mitochondrial function serve as important candidate genes in studying the heritability of compliance with and response to exercise training. One antagonist of mitochondrial function—ceramide—is a central compound in sphingolipid metabolism. Intracellular ceramide content is increased in obese individuals; and the accumulation of ceramide in non-adipocytes is implicated in many obesity-associated diseases: lipotoxic heart disease, atherosclerosis, and type 2 diabetes (Zhou et al., 2000; Birbes et al., 2002; Hannun and Obeid, 2002; Summers, 2006). Ceramide content is reduced with short exercise bouts and exercise training, although exercise effects on ceramide pathway components are complex and incompletely understood (Dobrzyn and Gorski, 2002a,b; Helge et al., 2004; Bruce et al., 2006; Baranowski et al., 2008; Błachnio-Zabielska et al., 2008, 2011; Bergman et al., 2016). Ceramide impinges on mitochondrial adaptations to exercise by directly inhibiting the respiratory chain, activating apoptotic pathways converging on mitochondria, and promoting programmed cell death and inflammation (Birbes et al., 2002; Hannun and Obeid, 2002; Summers, 2006; Mao and Obeid, 2008); these bioactive effects can be regulated via conversion of ceramide to alternate metabolites, including degradation by acid ceramidase (Mao and Obeid, 2008). Despite the regulatory roles of acid ceramidase on ceramide effects impacting exercise, to the best of our knowledge no one has investigated acid ceramidase genetic variants in exercise responses. We hypothesised that acid ceramidase genetic variants might impact ceramide metabolism and other downstream metabolic responses to exercise; exercise capacity; and thereby influence exercise adherence. Here, using two well-described randomised controlled exercise trials, we capitalised on a 35% non-completion rate to investigate the association of single nucleotide polymorphisms (SNPs) in the acid ceramidase (EC 3.5.1.23) gene (*ASAH1*) with the ability to predict completion of and response to exercise training.

## Methods

### Subjects

Two independent exercise trial samples were available: STRRIDE I (NCT00200993) and STRRIDE II (NCT00275145). A complete description of the STRRIDE I design is published elsewhere (Kraus et al., 2001). In summary, subjects were: 40–65 years of age; sedentary (exercised less than once weekly); overweight or obese (BMI 25 to 35 kg/m2); had fasting hyperinsulinemia (>10 IU/mL) with mild to moderate lipid abnormalities (LDL cholesterol between 130 and 190 mg/dL or HDL cholesterol <45 mg/dL for women or 40 mg/dL for men). The study design was similar. STRRIDE II, subjects were: 18–70 years of age; similarly, overweight or obese; sedentary; and with mild lipid abnormalities (Table 1). All subjects provided verbal and written informed consent as approved by the of Duke University Investigational Review Board and ECU Investigational Review Board. Subjects meeting inclusion criteria were randomised to one of four exercise groups in each study. Those subjects with DNA available for genetics studies are studied in this report.

**Table 1:** Characteristics of Subjects in STRRIDE I and STRRIDE II (% (N) or mean ± SD).

	**STRRIDE I**		**STRRIDE II**		**Total**		**Gene Expression Subset**
	**Failed to Complete Study**	**Completed Study**	**Failed to Complete Study**	**Completed Study**	**Failed to Complete Study**	**Completed Study**	
	28 (67)	72 (172)	38 (93)	62 (153)	33 (160)	67 (325)	*n* = 60
Age (y)	51.3 ± 5.8	52.5 ± 4.8	46.5 ± 11.8	48.8 ± 10.2	48.5 ± 10.0	50.8 ± 8.6	51 ± 1.2
Gender^**^- men	40 (27)	55 (94)	35 (33)	44 (68)	37 (60)	50 (162)	50 (30)
Women	60 (40)	45 (78)	65 (60)	56 (85)	63 (100)	50 (163)	50 (30)
Race^**^- white	58 (39)	81 (139)	67 (62)	86 (131)	63 (101)	83 (270)	90 (54)
Black	42 (28)	19 (33)	33 (31)	14 (22)	37 (59)	(17) 55	10 (6)
Body mass Index (kg/m2)	30.8 ± 3.6	29.9 ± 2.9	30.6 ± 3.3	30.5 ± 3.3	30.8 ± 3.4	30.2 ± 3.1	30.6 ± 0.4
Minimum Waist	96.2 ± 10.7	95.4 ± 9.8	95.7 ± 10.2	96.4 ± 9.6	96.0 ± 10.4	95.9 ± 9.7	96.9 ± 1.2
Circumference (cm)							
Pre-intervention peak VO_2_ (mL/kg/min)	26.4 ± 6.1	27.9 ± 6.1	26.6 ± 8.8	27.5 ± 6.0	26.5 ± 6.4	27.7 ± 6.0	27.6 ± 0.7

** *p-values for two sample t-test (completers vs. non-completers) significant in STRRIDE I, STRRIDE II, and total*.

### Exercise training

In STRRIDE I, subjects were randomly assigned to one of four groups: (1) non-exercising control; (2) low volume/moderate intensity aerobic exercise, defined as a caloric equivalent of 12 miles/week at 40–55% peak oxygen consumption (peak VO_2_); (3) low volume/vigorous intensity, defined as the caloric equivalent of 12 miles/week at 65–80% peak VO_2_; and (4) high volume/vigorous intensity exercise, defined as the caloric equivalent of 20 miles/week at 65–85% peak VO_2_. Caloric equivalents were determined by the approximate energy expenditure during walking or jogging for a 90 kg person; however, actual exercise modalities included cycle ergometers, treadmills, and elliptical trainers. The subjects underwent a 2-month ramp period in which exercise intensity and duration were gradually increased until the appropriate regimen was reached; this was followed by an additional 6 months of exercise training.

In STRRIDE II, subjects underwent a 3-month control run-in period followed by an 8-month exercise intervention in one of four exercise groups: (1) low volume/vigorous intensity group, identical to the low volume/vigorous intensity group of STRRIDE I; (2) resistance training, in which subjects completed a regimen of three sessions per week during which nine resistance exercises were performed with eight to twelve repetitions at 70–85% of one repetition maximum weight; (3) low volume/vigorous intensity aerobic exercise plus resistance training, during which subjects completed the low volume/vigorous intensity aerobic training protocol in addition to the resistance training protocol; and (4) high volume/vigorous intensity aerobic training, identical to that of STRRIDE I.

For the statistical analyses in which the STRRIDE I and STRRIDE II datasets were combined, the groups were coded as follows: (1) STRRIDE I inactive controls; (2) STRRIDE I low volume/moderate intensity aerobic exercise; (3) STRRIDE I plus STRRIDE II low volume/vigorous intensity aerobic exercise groups; (4) STRRIDE I plus STRRIDE II high volume/vigorous intensity aerobic exercise groups; (5) STRRIDE II resistance training group; (6) STRRIDE II low volume/vigorous intensity aerobic exercise plus resistance exercise group. Demographics and physiologic characteristics of the groups are shown in Table 1.

### Biologic measures

All phenotypic measures were taken prior to initiation and at completion of the exercise intervention (Month 6 in STRRIDE I and Month 8 in STRRIDE II). Peak VO_2_ was measured using a graded treadmill exercise testing protocol with gas exchange analysis (Duscha et al., 2005). Exercise compliance was measured as a percentage of the assigned exercise minutes per week completed by the subject averaged over the intervention period. Non-completion occurred when a subject withdrew from further participation in the study for any of the following reasons: time constraints; injury or illness unrelated to the study; medical problems; family issues; or geographic relocation. No subjects were lost to follow- up.

### Genotyping

DNA was isolated from whole blood using a commercial DNA isolation kit and a standard protocol (Qiagen, Inc, Valencia, CA). Acid ceramidase (*ASAH1*) SNPs were selected using the SNPSelector program in which a tagging algorithm prioritised SNPs for low linkage disequilibrium in the HapMap database, allelic frequencies, and regulatory potential (Xu et al., 2005). Six haplotype tagging SNPs in *ASAH1* were identified: rs7844023, rs2898458, rs7508, rs3810, rs2427746, and rs1049874; genotypes were determined using the Taqman assay (Applied Biosystems, Foster City, CA). The Taqman genotyping reaction was then amplified using a GeneAmp PCR system 9700 (95°C for 10 min, then 50 cycles at 92°C for 15 s, 60°C for 1 min). Fluorescence was detected using the 7900HT Taqman sequence detector (Applied Biosystems). Two reference controls were included. All SNPs were successfully genotyped for 95% or more of the individuals in the study; rescreening of 2.4% of subjects gave 100% identical results. Error rate estimates for SNPs meeting the reference control benchmarks were <0.2%.

### Gene expression profiling

We conducted gene expression analysis on a subset of 60 representative individuals randomly selected for further study from both data sets. The gene expression subgroup included 10 subjects (five men and five women) from each of the six exercise groups described above; paired baseline and post-training samples were always processed in the same assay. Demographics for this cohort were not significantly different from those of the entire cohort (Table 1).

Total RNA was extracted using the standard Trizol (Invitrogen, Carlsbad, CA) method and 30 to 50 mg of starting skeletal muscle. Two round amplification of total RNA was performed using a commercially available kit (Affymetrix, Santa Clara, CA). Thirty micrograms of biotinylated cRNA from each sample was hybridised to Affymetrix U133 Plus 2.0 microarrays. More detailed methods associated with microarray gene expression analysis can be found elsewhere (Hittel et al., 2005). Probe set expression levels were generated using the PLIER algorithm (typically 6 iterations) in Expression Console (Affymetrix) and imported directly into Partek Genomics Suite (Partek Inc., St. Louis, MO) for statistical processing.

### Statistical analysis

*Haploview* (27) was used to assess LD between SNPs using the combined STRRIDE I and STRRIDE II datasets. Genotype association with the non-completion study was analysed using a logistic regression model (SAS software, SAS Institute, Cary, NC). STRRIDE I and STRRIDE II datasets were analysed separately. As independent datasets, they provided an opportunity for validation of significance of individual SNPs in direction and magnitude of effect: STRRIDE II was considered the testing/exploratory set, and STRRIDE I the validation set. The outcome of non-completion of the exercise intervention was defined as a dichotomous variable with individuals who did not complete the intervention coded as 1; individuals who completed the program were coded as 0. Genotypes were coded using an additive model with 0, 1, or 2 copies of one allele and using a dominant model for the presence vs. absence of the same allele. Race, gender, and exercise group were included in the regression model; these variables differed significantly in those subjects completing the study vs. non-completers (Table 1). Due to the very small numbers of individuals reporting Asian or Hispanic ethnicity, only subjects who were either black or white were included in the analysis. To examine the potential for confounding by race, the logistic regression analyses were also performed stratified by race. Odds ratios were estimated for non-completion vs. completion.

Multivariable linear regression models (SAS software, SAS Institute, Cary, NC) were used to model genotype association with peak oxygen consumption (peak VO_2_) and exercise compliance in completers. Genotypes were coded for additive and dominant models. As described above, all models included terms for gender, race, and exercise group. Given the consistency of the effects observed for the SNPs associated with non-completion between STRRIDE I and STRRIDE II, the datasets were combined for analysis of change in peak VO_2._ Peak VO_2_ improvement models were tested with the complete dataset and with a dataset that excluded those subjects in the control and resistance training groups. Results are presented as mean ± SE.

Expression profile statistical analysis used Partek Genomics Suite (Version 6.4). Expression profiles were analysed to test differences in baseline gene expression between genotype groups. Following the gene expression value normalization, we used analysis of variance (ANOVA) with sex and race as covariates to examine genotype influences on mRNA expression also known as an eQTL analysis. *P* < 0.01 were considered statistically significant in this analysis.

## Results

### Allele frequencies

**Figure 1:**
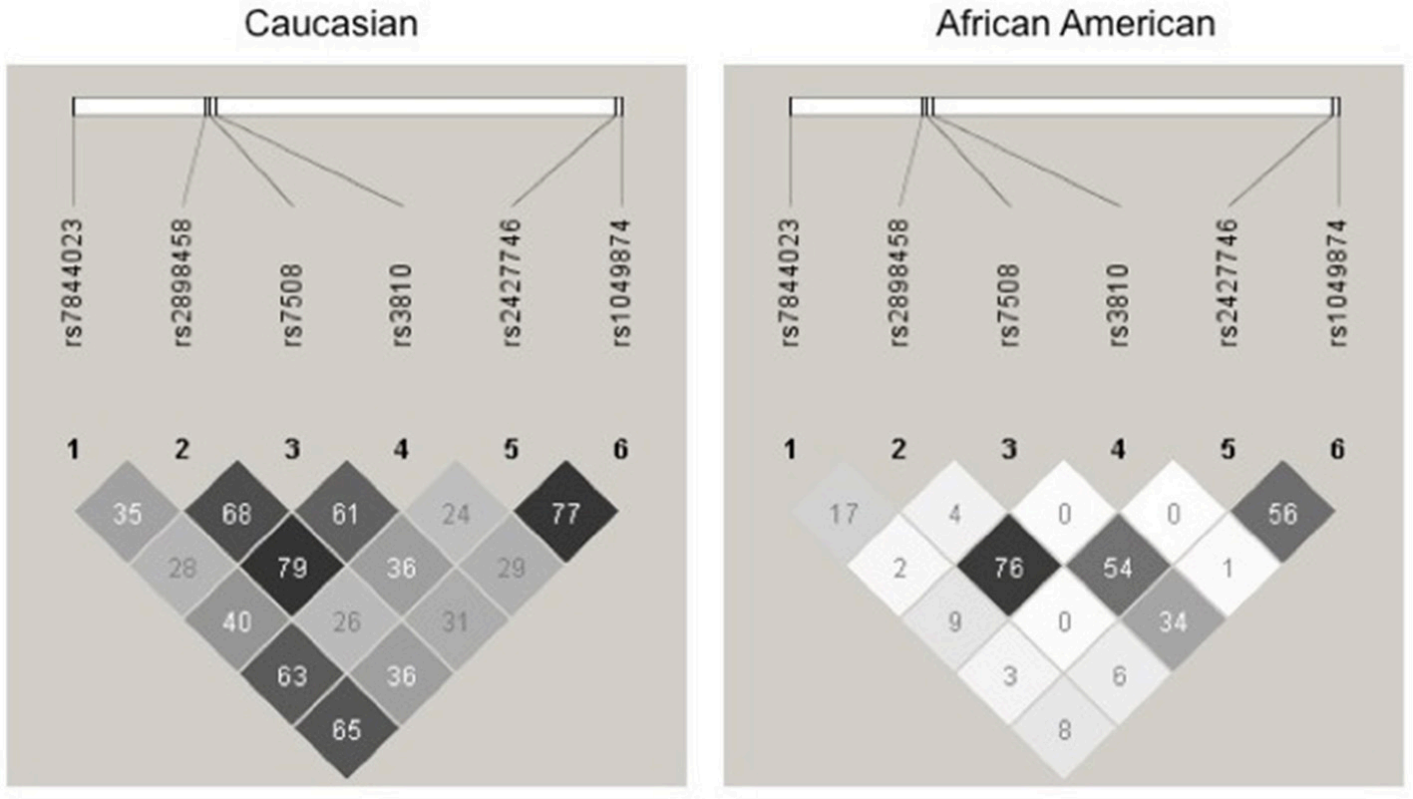
Linkage disequilibrium plot for Acid Ceramidase SNPs. SNPs assessed in the current study are shown above the linkage disequilibrium (LD) plots for genotype in white subjects (**left**) and black subjects (**right**) participating in either STRRIDE I or STRRIDE II. Results given are *r*^2^-values for association. The intensity of the block is proportional to the association between two variants. SNPs rs2898458, rs7508, and rs3810 as well as rs2427746 and rs1049874 are shown to be in LD(*r*^2^ > 0.6) in whites (*n* = 371). Only rs2898458 and rs3810 are in LD (*r*^2^ > 0.6) in blacks (*n* = 114).

For a complete gene analysis of the acid ceramidase gene, six *ASAH1* SNPs were genotyped (rs7844023, rs2898458, rs7508, rs3810, rs2427746, rs1049874) in 239 subjects in STRRIDE I and 246 subjects in STRRIDE II. All allele frequencies were in Hardy Weinberg equilibrium (chi^2^ test, *P* > 0.05) except for rs1049874 (Table 2). The rs1049874 SNP met all quality control benchmarks for genotyping as evaluated by an independent lab supervisor; it was therefore included in the analysis. Three SNPs, rs2898458, rs7508, and rs3810 were in pair-wise LD in whites (*r*^2^ > 0.60). Only SNPs rs2898458 and rs3810 were in strong LD in blacks (*r*^2^ >0.70; Figure 1).

**Table 2:** Genotype Frequency by Race.

**SNP**	**Genotype**	**STRRIDE I (*****N*** = **239)**		**STRRIDE II (*****N*** = **246)**	
		**White**	**Black**	**White**	**Black**
RS7844023	CC	26.5	25.9	24.5	30.8
	CT	46.5	46.6	53.2	40.4
	CT	27.0	27.6	22.3	28.8
	T Allele frequency	50.3	50.8	48.9	49.0
RS2898458	AA	50.3	7.4	44.4	22.0
	AG	38.2	40.7	47.6	38.0
	GG	11.5	51.9	8.0	40.0
	G Allele frequency	30.6	72.2	31.7	59.0
RS7508	AA	59.4	81.0	53.7	90.4
	AG	32.9	13.8	40.5	9.6
	GG	7.7	5.2	5.8	0.0
	G Allele frequency	24.1	12.1	26.0	4.8
RS3810	GG	45.3	7.1	43.1	11.8
	GT	41.8	39.3	48.4	45.1
	TT	12.9	53.6	8.5	43.1
	T Allele Frequency	33.8	73.2	16.2	65.7
RS2427746	AA	35.5	63.0	27.5	70.6
	AG	41.4	29.6	49.7	27.4
	GG	23.1	7.4	22.8	2.0
	G Allele Frequency	43.8	22.2	47.6	15.6
RS1049874	CC	29.0	12.1	23.0	54.9
	CT	36.7	31.0	51.3	37.2
	TT	34.3	56.9	25.7	7.8
	C Allele Frequency	47.3	27.6	51.3	26.5

### Genetic association with non-completion: exploratory analysis—STRRIDE II

**Figure 2:**
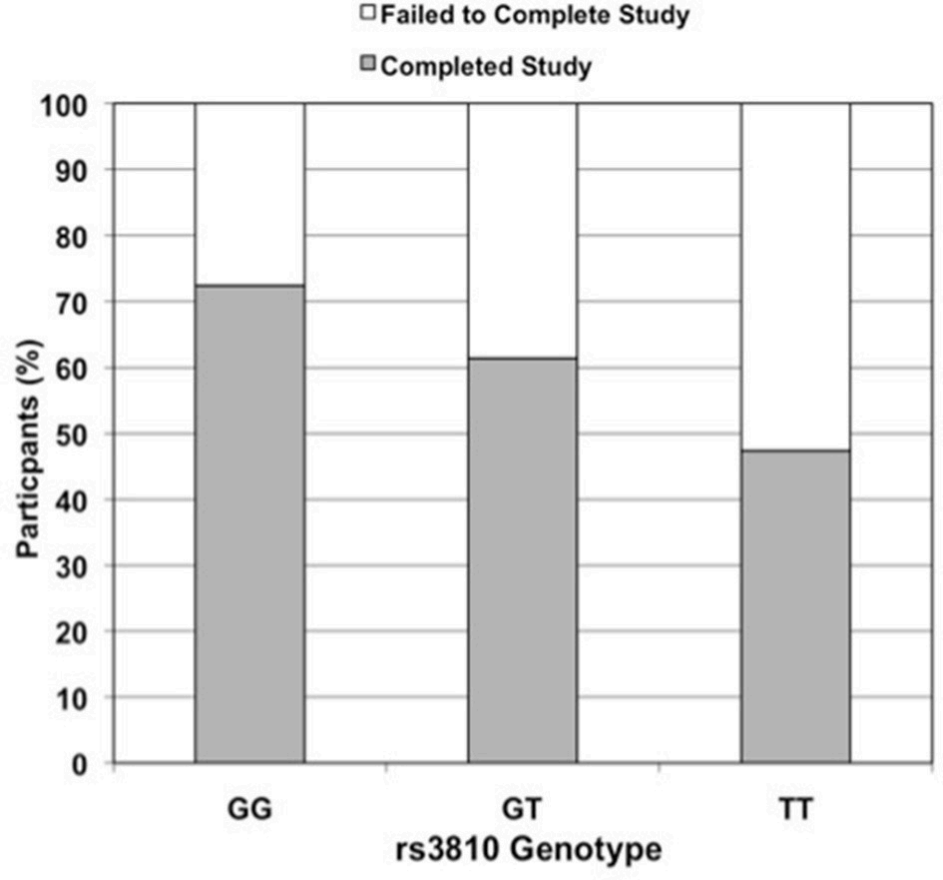
Effect of rs3810 genotype on completion rate in STRRIDE II. Percent of subjects in STRRIDE II completing (grey filled bars) and failing to complete (open bars) the study intervention by rs3810 genotype (GG genotype *n* = 87, GT genotype *n* = 114, TT genotype *n* = 38; when controlled for race, gender, and exercise group effects, additive model OR = 2.0, *p* = 0.01 and dominant model OR = 3.5, *p* = 0.005). These findings were similar to those for rs2898458 and rs7508 (data not shown).

Three *ASAH1* SNPs, rs2898458, rs7508, and rs3810—in LD—were consistently associated with intervention non-completion in both additive and dominant models (Table 3). After controlling for group, gender, and race, each additional T allele at rs3810 doubled the odds of failure to complete; in the dominant model, subjects with either one or two copies of the T allele were 3.5 times more likely to fail to complete than those with the GG genotype (additive model *P* = 0.01; dominant model *P* = 0.005; Figure 2). Similarly, both rs2898458 allele G and rs7508 allele G significantly increased the odds of non-completion in both additive (OR = 1.8, 1.8; *P* = 0.03, 0.002, respectively) and dominant (OR = 2.5, 2.6; *P* = 0.02, 0.02, respectively) models.

**Table 3:** Genotype association with risk of failure to complete study, controlling for race, gender, and intervention group.

		**Additive Model**		**Dominant Model**	
		**Odds Ratio**	***P*-value**	**Odds Ratio**	***P*-value**
rs7844023 (C/**T**)^*^	STRRIDE I	0.88	0.562	0.88	0.300
	STRRIDE II	0.86	0.513	0.82	0.596
rs2898458 (A/**G**)^*^	STRRIDE I	1.80^*^	0.022^*^	1.80	0.384
	STRRIDE II	1.79^*^	0.026^*^	2.52^*^	0.018^*^
rs7508 (A/**G**)^*^	STRRIDE I	1.17	0.546	1.17	0.499
	STRRIDE II	1.78^*^	0.034^*^	2.55^*^	0.015^*^
rs3810 (G/**T**)^*^	STRRIDE I	1.79^*^	0.016^*^	1.79	0.687
	STRRIDE II	2.02^*^	0.012^*^	3.49^*^	0.005^*^
rs2427746 (A/**G**)^*^	STRRIDE I	0.95	0.838	0.95	0.455
	STRRIDE II	1.17	0.543	1.15	0.711
rs1049874 (T/**C**)^*^	STRRIDE I	1.11	0.635	1.11	0.92
	STRRIDE II	1.18	0.628	1.21	0.628

All models controlled for race, gender and intervention group. Significant effects are marked with asterisk

(*) *and indicated in bold*.

### Genetic association with study non-completion: validation analysis—STRRIDE I

**Figure 3:**
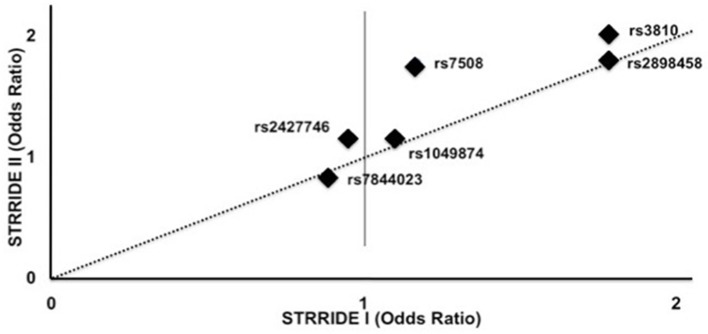
Genotype effect on odds of failure to complete study by SNP in STRRIDE II vs. STRRIDE I. Additive model odds ratios for risk of study non-completion for each ASAH SNP for STRRIDE I (x axis) and STRRIDE II (y axis). Two SNPs significantly increased the odds of failure to complete both STRRIDE II and STRRIDE I: rs2898458 and rs3810. SNP rs7508 significantly increased the odds of study non-completion in STRRIDE II.

*ASAH1* genotype associations were validated in the STRRIDE I dataset, in which the additive model was significantly associated with non-completion for rs3810 and rs2898458 (OR = 1.8, 1.8; *P* = 0.02, 0.02). When comparing the direction and magnitude of association of each SNP, the two independent datasets showed excellent agreement, providing strong replication for association of these SNPs with failure to complete (Table 3, Figure 3). The race-stratified analyses supported the combined analysis with the white race group showing results consistent with the analysis of the full datasets for both STRRIDE I and STRRIDE II (supplemental tables).

### Genetic association with peak oxygen consumption in intervention completers

To investigate potential mediators of the genetic association with failure-to-complete an exercise intervention, we studied the effects of the *ASAH1* variants on baseline peak VO_2_ and change in peak VO_2_ with exercise training; we hypothesised that poor exercise capacity or ability to respond to exercise training might explain why persons chose to not complete the protocol. Baseline peak VO_2_ was not significantly associated with genotype or study completion (data not shown). However, among completers, improvement in peak VO_2_ was correlated with genotype for two of the three *ASAH1* SNPs previously associated with non-completion. When compared to subjects homozygous for the G allele for SNP rs3810 and controlling for race, gender, group, and baseline peak VO_2_, each additional T allele decreased the improvement in peak oxygen consumption by 1.4 (mL/kg/min) (*p* = 0.0185, *r*^2^ = 0.3689). Similarly, the G allele of SNP rs2898458 showed a smaller improvement in peak VO_2_ (0.45 mL/kg/min decrease, *P* = 0.0734, *r*^2^ = 0.362).

**Figure 4:**
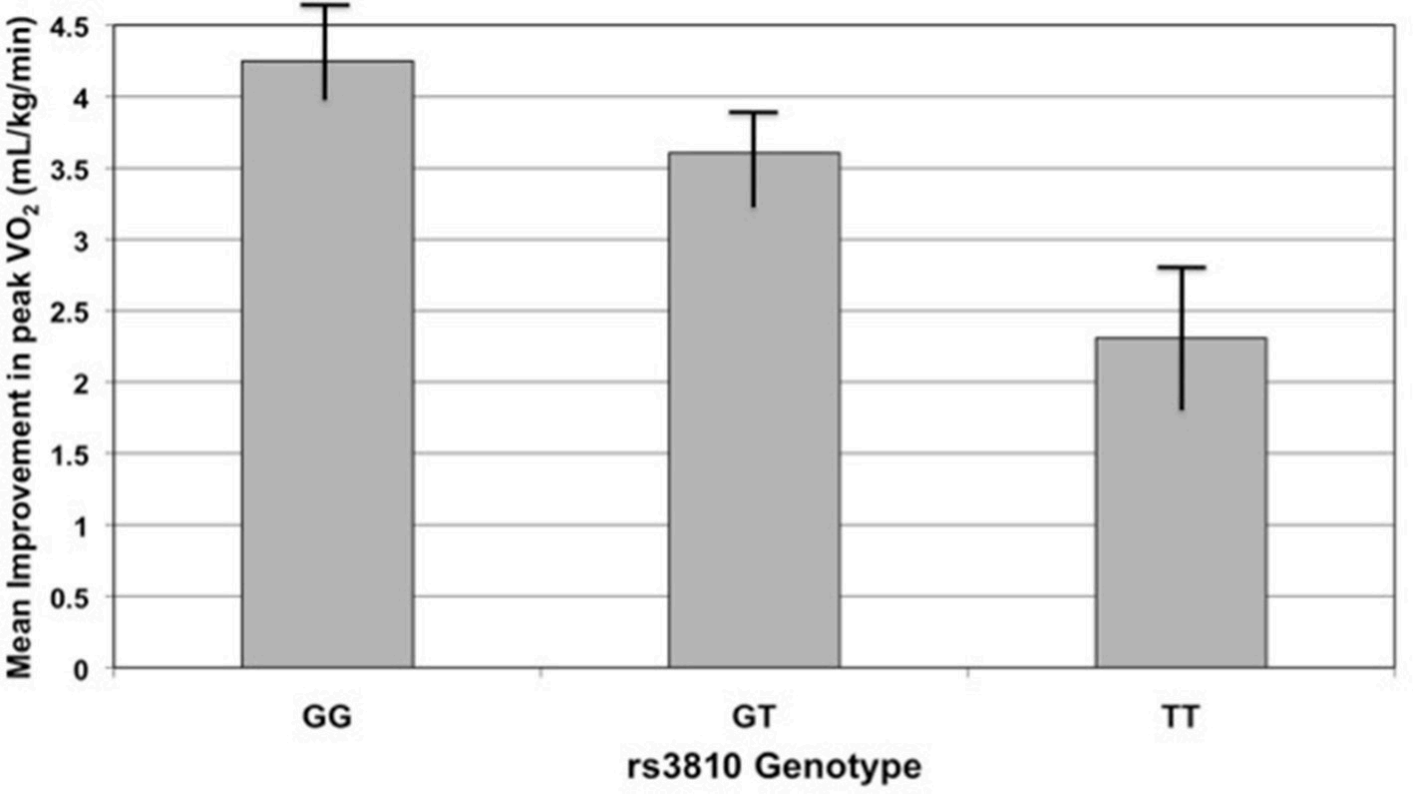
Improvement in peak oxygen consumption (peak VO_2_) with exercise training by rs3810 genotype. Improvement in peak VO_2_ (mL/kg/min), measured as the difference in peak VO_2_ before and after an exercise intervention, compared to rs3810 genotype. STRRIDE I and STRRIDE II datasets were combined for the analysis (GG genotype *n* = 168, GT genotype *n* = 207, TT genotype *n* = 90). The rs3810 genotype significantly predicted change in peak VO_2_ (*p* = 0.0185), despite the fact that some groups differed in the intensity of the training stimulus.

One would not expect peak VO_2_ to change significantly in those subjects assigned to either the control or resistance training groups; we therefore tested the association of improvement in peak VO_2_ with genotype in the dataset excluding these two groups. Indeed, the two SNPs mentioned previously remained correlated with improvement in peak VO_2_ (rs3810 *r*^2^ = 0.29, *P* = 0.036; rs2898458 *r*^2^ = 0.29, *P* = 0.0834; Figure 4). In this model, rs7508 genotype was also associated with improvement in peak VO_2_ (*p* = 0.0873, *r*^2^ = 0.28).

### Genetic effects on mRNA expression of ASAH1

**Figure 5:**
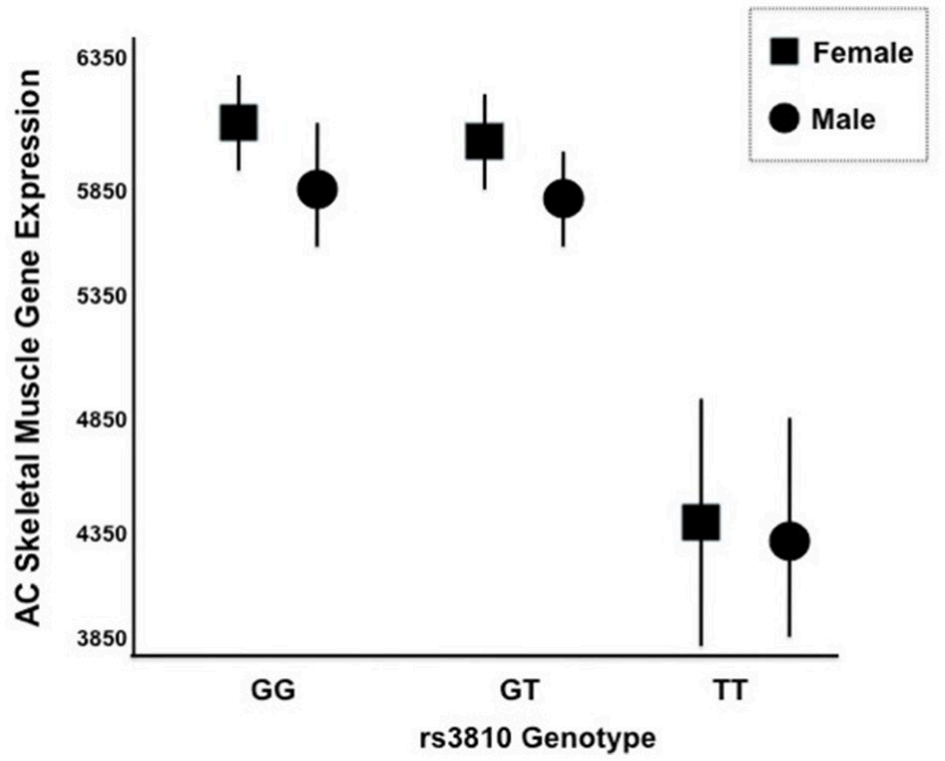
mRNA Expression of ASAH1 Stratified by rs3810 Genotype. Two distinct Affymetrix probe sets indicated that ASAH1 mRNA expression is significantly different by rs3810 genotype using either full (*p* = 0.006–0.007) or dominant (*p* = 0.01–0.02) models, using sex and race as covariates. Females are represented by rectangles and males are represented by circles. TT individuals demonstrated 1.3–1.4 times lower skeletal muscle acid ceramidase expression than did GG individuals at baseline.

We generated gene expression profiles using a subset of 60 individuals from both STRRIDE I and STRRIDE II. As shown in Table 1, demographic data for age, height, weight, and BMI were not different between the total cohort and the gene expression-profiled sub-cohort. Peak VO_2_ at baseline was lower in the gene expression subgroup than the total population; however, it was not different between the subgroup and all subjects that completed the study. There are five probe sets for *ASAH1* on the U133 Plus 2.0 microarray, of which only two (213702_x_at and 210979_at) target the full length *ASAH1* transcript and are highly expressed in skeletal muscle. Expression of these two probe sets was significantly different between rs3810 genotypes, when tested with either an additive (*p* = 0.006 and *p* = 0.007 for probe sets 213702_x_at and 210979_at, respectively) or a dominant model (*p* = 0.00007 and *p* = 0.0001 for probe sets 213702_x_at and 210979_at, respectively). For rs3810, the TT group demonstrated between a 1.3 and 1.4 times reduction in *ASAH1* mRNA expression when compared with the GG group (Figure 5). Linkage disequilibrium between rs3810 and rs2898458 was nearly complete in the gene expression subgroup. Therefore, similar results were found for the relation between skeletal muscle *ASAH1* expression and rs2898458 genotype (data not shown). Linkage disequilibrium between rs3810 and rs7508 was somewhat lower at 78%, with only one GG subject (also homozygous for the minor allele for both other SNPs); however, the gene expression results for rs7508 were similar to those for rs3810 (not shown).

## Discussion

In the present study, we provide evidence suggesting that genetic variation within the acid ceramidase gene is associated with an individual's ability or willingness to persist in a newly prescribed exercise program. Three of six *ASAH1* SNPs (rs2898458, rs3810, and rs7508; all in LD) were associated with significantly increased risk of non-completion of an exercise intervention in two exercise training interventions — STRRIDE I and STRRIDE II. These SNPs were also associated with change in peak VO_2_ among the completers; there also were differences in *ASAH1* gene expression such that genetic correspondence with gene expression was exactly consistent with less improvement in VO_2_ with training and greater non-completion rates. Thus, these findings support plausible biological links between genetic variation in *ASAH1* and exercise behaviour.

Ceramide is produced either via hydrolysis of membrane sphingomyelin; or *de novo* from long- chain saturated fatty acids. In non-adipocytes, increased fatty acid deposition with subsequent conversion to ceramide has been proposed as a mechanism of lipotoxicity in type 2 diabetes, heart failure, and atherosclerosis (Zhou et al., 2000). Accumulation of ceramide initiates pro-apoptotic pathways converging on mitochondrial function—the major source of pro-apoptotic molecules (e.g., BCL1). (Birbes et al., 2002). Increased intracellular ceramide leads to the apoptosis of pancreatic beta cells and cardiac myocytes in obese rats leading to decreased function of both cell types (Unger et al., 1999; Zhou et al., 2000). Ceramide also directly inhibits both complex I and complex III of the mitochondrial respiratory chain in rat heart muscle *in vitro* (Gudz et al., 1997; Di Paola et al., 2000).

Acid ceramidase, ubiquitously expressed in somatic cells, plays a crucial role in the maintenance of cellular ceramide concentrations (Park and Schuchman, 2006). Acid ceramidase metabolizes ceramide into sphingosine and free fatty acids. Over-expression of the enzyme prevents the inhibitory effects of accumulated saturated fatty acids on insulin signalling(Chavez et al., 2005) while abnormally high expression of the enzyme has been reported in several human cancers (Park and Schuchman, 2006). Ceramide content is increased in skeletal muscle of obese, insulin resistant humans (Adams et al., 2004) and endurance exercise reduces the content of ceramide in skeletal muscle in obese subjects with a concomitant improvement in insulin sensitivity (Bruce et al., 2006). Nonetheless, ceramide relationships to insulin sensitivity and changes with exercise are complex and incompletely understood: effects appear dependent on ceramide species, exercise duration and intensity, and underlying insulin sensitivity (Dobrzyn and Gorski, 2002a; Helge et al., 2004; Baranowski et al., 2008; Błachnio-Zabielska et al., 2008; Bergman et al., 2016). Further, in addition to acid ceramidase, ceramide regulation can occur via degradation by neutral and alkaline ceramidases or conversion to sphingomyelin.

It is important to recognize that we have not necessarily found functional acid ceramidase variants; rather, we only have identified and replicated associations implicating the acid ceramidase gene in the biological response. Nonetheless, in light of recognised acid ceramidase functions, it is intriguing that acid ceramidase polymorphisms were associated with reduced completion rates for and poorer peak oxygen consumption (peak VO_2_) responses to exercise training. All three significant acid ceramidase SNPs were associated with differential improvements in peak VO_2_ with exercise. While only one of the SNPs was significant in this association, one should note that post-intervention peak VO_2_ measurements were available for exercise completers only. Were it feasible to include both exercise completers and non-completers in the post-intervention peak VO_2_ analysis, the correlation between acid ceramidase minor allelic genotype and decreased improvement in oxygen consumption may have been stronger for all three genotypes.

There were significant differences in racial groups for non-completion rates, as well as large differences in allele frequencies for rs3810 and rs2898458; this raised the issue of the potential for allele frequency differences to confound the strong association observed for these variants. To evaluate the stability of the associations observed in the overall group, we performed race-stratified analyses for each study. In both whites and blacks, rs3810 demonstrated association with non-completion; the odds ratios for the same alleles in both races and both studies were very consistent. The non-significant *p*-values in the smaller black subgroup reflected differences in allele frequency and sample size. Although detailed evaluation of genetic variation across *ASAH1* will be required to identify functional SNPs, comparing the results for blacks and whites in light of the expected differences in linkage disequilibrium patterns provided specific support for rs3810 as the SNP of interest in *ASAH1*.

We routinely queried participants for their reasons when withdrawing from the study prior to completion. The reasons varied considerably: among others were time constraints; family issues; “a changed mind”; or unrelated medical problems. Clearly, many issues can affect compliance with behavioural interventions—including exercise—not explainable by genetics alone. However, the association between study non-completion and acid ceramidase genotypes remained significant despite the “noise” created by the subjects' social environment and personality traits; this implies that the biological relation might be even stronger than our findings indicate. Furthermore, the association of the phenotypic measure of peak VO_2_ improvement with exercise compliance and acid ceramidase genotype points to a physiologic mechanism for exercise intolerance. Perhaps the genetically associated unresponsiveness to exercise training may consciously or subconsciously play into the willingness of individuals to continue to participate in an exercise program; this may be exacerbated by personality factors, life stress, or other environmental influences.

Personality characteristics may play into the interplay between genetics and exercise behaviour (Herring et al., 2014). Elements of the Big Five Personality Factors are associated with other lifestyle elements—dietary habits and smoking, among others—and also are associated with aerobic capacity (Terracciano et al., 2013) muscle strength (Tolea et al., 2012) and adherence to post-surgery rehabilitation (Hilliard et al., 2014). It would be important—and perhaps therapeutically useful—to know whether the half of individuals with the “drop-out genotype” that persisted with the intervention have a different personality profile than those that fail to complete the intervention. If genetic effects on exercise behaviour are mediated—at least in part—through personality factors; and if personality factors explain the variation in exercise adherence behaviour among those that are at genetic risk of poor adherence; then one might want to test whether personality factors can inform personalised strategies and messaging to increase adherence for those whose health would most benefit from increases in regular exercise.

To our knowledge this is the first report of the effects of acid ceramidase polymorphisms on exercise behaviour in overweight to mildly obese, insulin resistant subjects. Our findings should be validated in subsequent studies. Given resource constraints and sample availability, we were unable to quantify acid ceramidase enzyme activity, *per se*, within our subjects. We also did not measure whether acid ceramidase gene variants differentially affected skeletal muscle enzyme or ceramide content in these subjects—either prior to or in response to an exercise intervention. Future studies will be necessary to further elucidate the relation between common gene variants of acid ceramidase with skeletal muscle enzyme activity, ceramide content, and physical performance.

In conclusion, these data suggest that genetic variation within the acid ceramidase gene significantly affects exercise tolerance and completion of an exercise program. Acid ceramidase regulates ceramide content within skeletal and cardiac muscle; these effects are likely to be involved in muscle adaptations to exercise training. Due to the proven health benefits of regular exercise, characterization of individual exercise potential conferred by genetic variation prior to initiation of an intervention may be helpful in maximizing the adherence to exercise and therefore the health benefits accrued therefrom.

## Ethics statement

Informed consent was obtained under protocols approved by the Investigational Review Boards of Duke University and East Carolina University.

## Author contributions

WK and MD conceived the science. WK and EPH financial support for the research. WK and LL wrote manuscript. WK, CS, JH, LL, ICS, and MH conducted experiment. LL, ERH, and MH performed analysis. WK, KH, and ICS edited manuscript.

### Conflict of interest statement

The authors declare that the research was conducted in the absence of any commercial or financial relationships that could be construed as a potential conflict of interest.
